# Nanotechnology-based approaches for the removal of microplastics from wastewater: a comprehensive review

**DOI:** 10.3762/bjnano.16.114

**Published:** 2025-09-15

**Authors:** Nayanathara O Sanjeev, Manjunath Singanodi Vallabha, Rebekah Rubidha Lisha Rabi

**Affiliations:** 1 Department of Civil Engineering, Mepco Schlenk Engineering College, Sivakasi - 626005, Tamil Nadu, India; 2 Department of Civil Engineering, B.M.S. College of Engineering, Bangalore, 560019, Karnataka, India

**Keywords:** artificial intelligence, membrane technology, microplastic, nanotechnology, nanoadsorbents, nano robots, photocatalysis

## Abstract

The increasing prevalence of microplastics (MPs) in aquatic environments has raised significant concerns due to their persistence, potential for bioaccumulation, and adverse effects on human and ecosystem health. Conventional wastewater treatment technologies are largely inadequate for effectively removing MPs, especially those in the nanosize range. This review presents a detail analysis of the sources, pathways, detection methods, and health impact of MPs, while emphasizing the emerging role of nanotechnology in their remediation. Nanomaterials, including nanoadsorbents, photocatalysts, and advanced membrane materials, exhibit unique properties such as high surface area, enhanced reactivity, and tunable surface chemistry, which offer promising avenues for the selective and efficient removal of MPs from water. This paper also explores the mechanism, performance and limitations of various nanoenabled treatment strategies such as adsorption, photocatalysis, and membrane filtration using materials like metal-organic frameworks, carbon-based nanomaterials, MXenes, and metal oxides. It also highlights recent innovations such as microrobotic systems and AI-assisted detection frameworks for MP monitoring. Despite high laboratory scale efficiencies, there are several challenges such as material scalability, environmental safety, regulatory frameworks, and real water applicability. This study proposes future directions for sustainable nanotechnology deployment, including green synthesis, hybrid system integration, and machine learning optimization. Together, these approaches aim to establish a comprehensive, scalable, and environmentally safe solution for the remediation of MPs in wastewater systems.

## Introduction

Plastic pollution has become a crucial environmental concern recently. It was reported that from 1950 to 2015, global plastic production increased from 5 to over 300 megatons, with approximately 60 to 99 megatons turning into waste. It is estimated that, by 2060, the plastic waste generation could increase annually to 155–265 megatons. In spite of the continuous increase in the production of plastic, the rate of recycling remains low worldwide [1]. Microplastics (MPs) are tiny debris pertaining to plastic of size less than 5 mm. They are classified based on size, origin, and polymeric composition [2]. Morphologically, MPs appear as foam, beads, sheets, fragments, and fibres, with fibres being the most prevalent type, often originating from wastewater discharged by the textile industry. Based on their origin, MPs are divided into two groups, that is, primary and secondary. Primary MPs are intentionally produced in small sizes for the use in a range of products like cosmetics, clothing, and personal care items. In contrast, secondary MPs are formed when larger plastic items break down due to environmental factors like sunlight, physical wear, and microbial activity [3]. Common types of MPs found in ecosystems, particularly in freshwater and drinking water sources, include materials such as high-density polyethylene, polystyrene, polyvinyl chloride, and polypropylene [4].

Among the various contributors to MPs, residential households play a major role. Everyday plastic items ranging from toothbrushes over kitchenware to furniture, such as plastic chairs that are commonly used in homes, contribute to MPs release [5]. Wastewater treatment plants receive MPs primarily from two sources, namely, domestic sewage and industrial effluents. In domestic sewage, MPs typically originate from personal care and cosmetic products, as well as from laundering synthetic textiles. In contrast, industrial wastewater contains MP that are largely generated through the wear and tear of larger plastic items throughout their production, usage and disposal stages [6].

The tiny MP particles can directly harm marine life through ingestion or indirectly by attracting and accumulating environmental pollutants [7]. Experimental studies show that exposure to MPs can result in a wide range of harmful effects such as disruptions in metabolism, oxidative stress, immune system activation, developmental and reproductive toxicity, and damage to the nervous system [8].

MPs possess high specific area and strong adsorption capacity, enabling them to attract pollutants from the environment. They can accumulate harmful substances such as polycyclic aromatic hydrocarbons, heavy metals, and other toxic contaminants, increasing their potential risks to living ecosystems and organisms [9–10]. MPs have low density, variable sizes, high persistence, and non-biodegradable nature. These characteristics make their removal difficult, especially in aquatic environments [11]. Several studies have measured the presence of MPs in commonly consumed products such as alcoholic beverages, tap and bottled water, seafood, and salt. Reported concentrations vary, ranging from 0.10 to 1.48 MPs per litre in food items and between 4.23 and 94.37 MPs per litre in beverages [12]. The source of these contaminants includes airborne particles introduced during food processing or handling, degradation of plastic packaging, and, most significantly, contamination from polluted freshwater sources [13].

Primary, secondary, and tertiary treatment methods have been explored to mitigate MP contamination. Meanwhile, a more sustainable solution remains essential for the future. In recent years, extensive research has focused on several processing technologies, including coagulation, advanced oxidation processes, microbial degradation, membrane bioreactor, rapid sand filtration, and dissolved air flotation [14–15].

**Figure 1:**
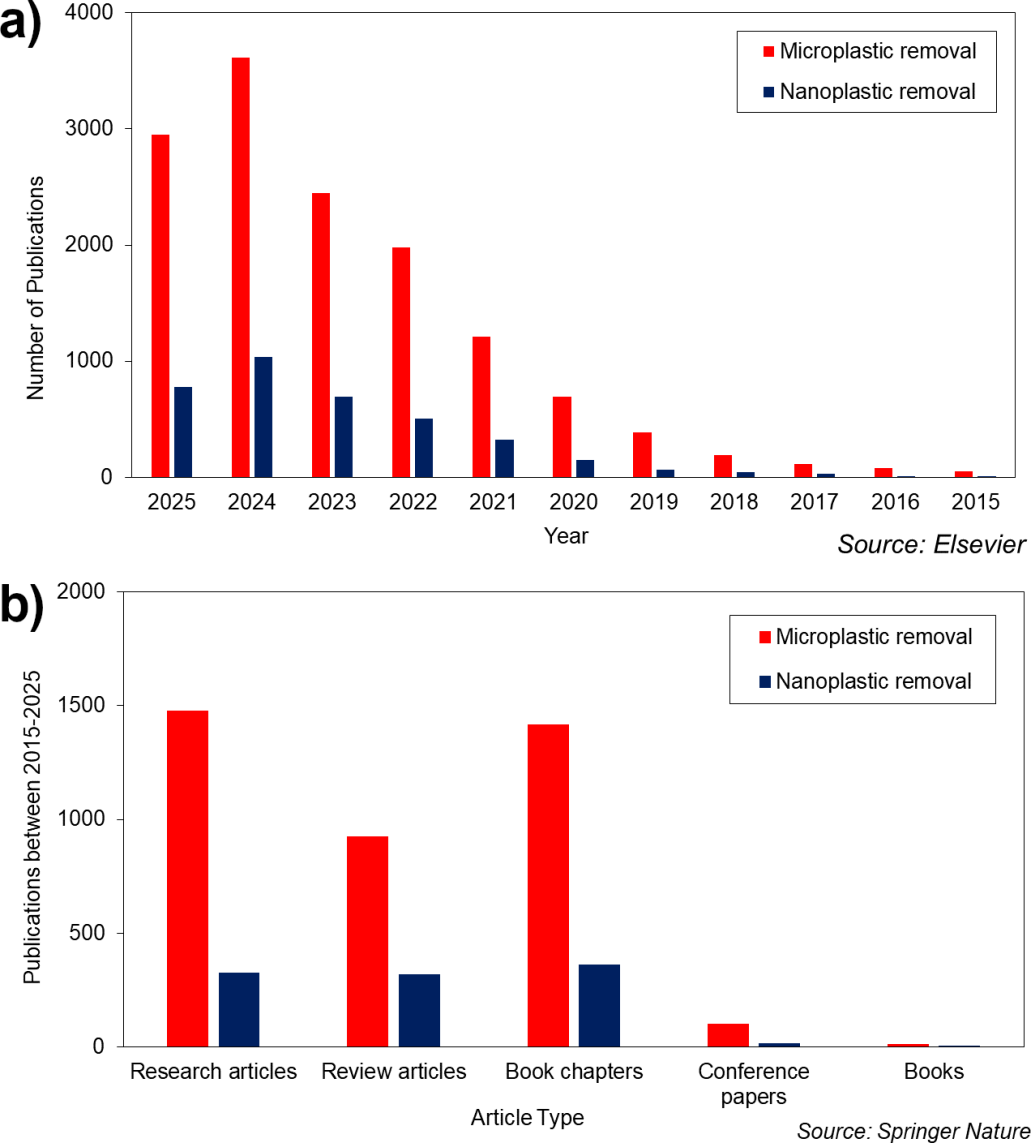
(a) Number of publications in the past decade with MP and NP removal keyword search from Elsevier, (b) number of publications in the past decade with MP and NP removal keyword search from Springer nature.

The publication trend over the past decade, as illustrated in Figure 1, demonstrates a significant rise in research activities focused on the removal of MPs and nanoplastics (NPs). Figure 1a, based on a keyword search from Elsevier’s database, reveals a consistent year-on-year increase in the number of publications related to MP removal from 2015 to 2025. Notably, the number of studies surged from just 80 in 2016 to a peak of 3616 in 2024. A similar trend, though less pronounced, is observed for NP removal, which also showed a steady growth, rising from 48 publications in 2015 to 1039 in 2024. This indicates a growing recognition of NPs as emerging contaminants, though they still receive less attention than MPs. In contrast, Figure 1b provides insights from Springer Nature, categorizing the type of publications related to both MP and NP removal between 2015 and 2025. Research articles dominate the landscape, with 1478 publications focused on MPs and 325 on NPs. Review articles and book chapters also show substantial number of MP studies, suggesting a mature and well-reviewed body of literature. In contrast, contributions on NPs remain limited across all categories, reflecting the relatively nascent stage of this research area. Together, these figures underscore a growing scientific interest in plastic pollution, with a marked focus on MPs. However, the emerging concern around NPs present an opportunity for further in depth research and exploration.

Advancements in synthesis and characterisation techniques have enabled the detection of materials at the nanoscale, unlocking new opportunities across various streams. In the field of water treatment, nanotechnology is gaining wide attention due to its enhanced efficiency, effectiveness, affordability, and durability. The key properties of nanoparticles include high surface area, extensive functionalization, high reactivity, and size-dependent characteristics. By leveraging these properties, water treatment methods can be refined at the nanoscale to selectively target pollutants [16–17].

This comprehensive review explores the potential of nanotechnology in removing MP contamination from water and wastewater. Unlike conventional treatment methods, which are less effective in capturing nanoscale plastic pollutants, nanotechnology-based approaches offer precision, enhanced adsorption, and catalytic degradation capabilities. By combining the principles of water treatment technologies and nanoscience, this study highlights innovative pathways for improved removal efficiency, selective pollutant targeting, and sustainable application. As the global concern over plastic pollution is a rising concern, this review sets the stage for the next generation of water treatment strategies, focusing on the application of nanotechnology for the production of safer and cleaner water resources.

## Review

### Source, pathway and detection of MPs in water and wastewater

**Figure 2:**
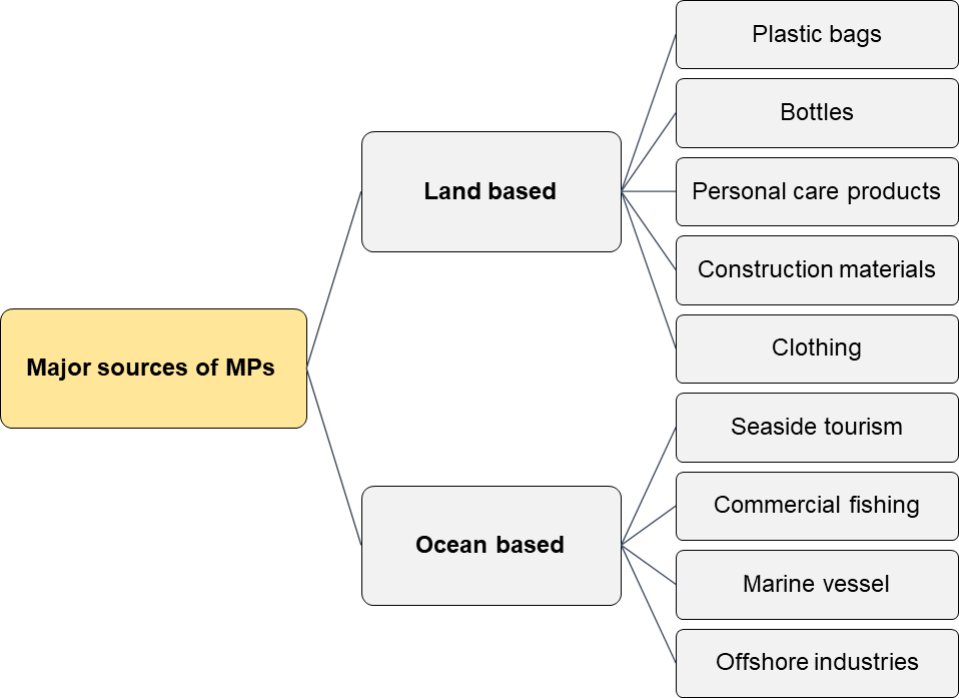
Different sources of MPs.

MPs are formed through primary processes and from secondary sources [18]. Figure 2 represents the different sources of MPs.

The marine environment is considered as the primary sink of MPs. MPs that are generated on land are eventually transported by various modes, including surface runoff and streams, and find their way to ocean. In oceans, these particles with low density are present initially in the suspended form. However, as time passes, they interact with suspended clay particles, and an accumulation of biofilm occurs, resulting in an increase in density. This will result in gradual settlement of the MPs in the sediment [19]. The concentration of MPs in wastewater varies, with levels reaching several hundred particles per litre [7]. Studies have reported significant variation in the concentration and types of MPs present in wastewater samples collected from different treatment plants. Commonly identified MPs include polyethylene (PE, 4–51%), polyester (PES, 28–89%), polystyrene (PS) (5–27%), polyethylene terephthalate (PET, 4–35%), polypropylene (PP), and polyamide (PA, 3–30%) [20]. In a study performed by Steinfield et al. [21], it was observed that the MP concentrations in untreated wastewater from paper mills range from 10^6^ to 10^8^ (MPs ≥ 20 µm/m^3^). The paper mills studied, manufacture a variety of paper products, including tissue paper, cardboard, and specialized items, using different raw materials and polymeric additives like polymeric fibres and coating colours. It was observed that, in paper mill waste, the polymeric additives are the predominant source of microplastic.

In a study performed by Liu et al. [22], it was observed that the petrochemical industry plays a major role in microplastic pollution. Crude oil undergoes extraction, refining, and cracking to yield low-molecular-weight monomers like ethylene and propylene. These monomers are later polymerised to produce various plastic materials. This results in the release of microplastic particles, which enter into wastewater treatment facilities through industrial effluents.

Wastewater treatment plants (WWTPs) are the major contributors to microplastic pollution in the environment. They serve as critical points for collecting and filtering MPs from domestic sewage, storm water runoff, and industrial effluents. However, conventional WWTPs often fail to completely remove MPs, leading to MP discharge into natural water bodies [23]. The primary sources of MPs in wastewater treatment plants include plastic-based industrial effluents, synthetic textile fibres from clothing, personal care products in household wastewater, wear and tear of road tires, and discharges from textile manufacturing [9]. Table 1 lists some of the MP sources in wastewater with the corresponding concentrations. In aquatic environments, MPs transform due to the mixing with aggregates, biofouling, and leaching of additives. These alterations affect their buoyancy, leading to their accumulation in benthic ecosystems [24].

**Table 1:** MP concentration and detection method in wastewater.

Source	MPs dimension	MPs detection method	Dominant microplastic type^a^	Concentration	Ref.

primary sludge and biosludge from paper and pulp industry	<20 μm	Raman spectroscopy	PE, PP	primary sludge: 900–1600 MPs/g dry weight biosludge: 210 MPs/g dry weight	[25]
WWTP in antarctica	<50 μm	micro-Raman spectroscopy	PP, PVC, PTFE, PET, PS	64 to 159 particles per litre	[26]
secondary WWTP located on the river Clyde, Glasgow	–	–	Microbeads	15.70 ± 5.23 particles per litre	[27]
drinking water treatment plant	0.5–0.1 mm and 5–1 mm	–	wastewater dominated by synthetic fibre (polyester type), while drinking water was characterised by fragments and fibres	2–11 particles/m^3^	[28]
WWTP of an organized industrial zone, Bursa	fibre, 500–1000 µm	–	PE, PY, PET, PP	480–801 particles/m^3^	[29]
inlet of WWTP Guheshwori, Kathmandu city, Nepal	fibre, fragments, foam, and pellets, 500–150 μm size	–	–	31.2 ± 17.3 particles per litre	[30]
hospital laundry wastewater Copenhagen, Denmark	100–200 μm	–	–	1.4 × 10^6^ particles per litre	[31]
reclaimed WWTP	–	Raman spectroscopy	–	0.75 ± 0.26 particles per litre	[32]
Kappala wastewater treatment plant in Sweden	–	FPA-μ-FTIR, ATR-FTIR, and stereoscopic microscope	–	6.42 × 10^10^ counts/day	[33]
effluent of WWTP, Portugal	–	μ-FTIR and microscope	–	52–233 particles per litre	[34]
WWTP, Denizili, Turkey	fibres in 100–500 µm	μ-FTIR and visual sorting	PE, PVA	140 particles per litre	[35]
influent of Farabi Hospital WWTP, Iran	<0.5 mm	SEM, FTIR, stereomicroscope	PP, PE, Latex, PU, PS, PA, Nylon, HDPE	23 particles per litre	[36]
sewage treatment plant, China	0.22 to 5.0 mm	TD-GC/MS	PE	Influent: 1313.11 ± 336.96 μg/L Effluent: 25.84 ± 3.75 μg/L	[37]
influent of M'zar WWTP located in Agadir metropolis	100–500 μm	stereoscopic microscope, ATR-FTIR and SEM-EDX	PE, PP and PS	519 MPs/L	[38]
influent of WWTP sited in Southwest Europe	>500 µm	stereomicroscopy and FTIR spectrophotometry	PE, PET and PP	16.1 ± 3.3 MPs/L	[39]
influent of WWTP in Danang, Vietnam	1.6 to 5000 µm	FTIR	PE, PET, Nylon and PVC	183–443 MPs/L	[40]
municipal WWTP in Thailand	0.05–0.5 mm	optical stereomicroscope and FTIR	PET, PE, PP	77 ± 7.21 MPs/L	[41]
sewage treatment plant, Bihar	<250 μm	optical microscope, FESEM-EDX and ATR-FTIR	LDPE	64.3 ± 4.89–47.66 ± 4.71 MPs/L	[42]

^a^PP – polypropylene, PVC – polyvinyl chloride, PTFE – polytetrafluoroethylene, PET – polyethylene terephthalate, PS – polystyrene, PE – polyethylene, PY – polyester, PU – polyurethane, PA – polyamide, HDPE – high-density polyethylene, LDPE – low-density polyethylene.

### Impact of MPs on health and ecosystems

In smaller organisms like plankton, MPs have severe impact because of their small size, widespread presence, and ability to absorb pollutants [43]. Once consumed, MPs may cause physical harm to organisms, restrict food intake and interfere with plankton feeding [44]. Additionally, they can also damage the digestive tract, potentially affecting plankton development, lifespan and reproduction, and growth [45]. MPs function as carriers for heavy metals, resulting in their accumulation in the digestive systems of fish. This accumulation can negatively affect fish by impairing their activity, stunting growth, disrupting reproduction, and potentially leading to mass mortality. Such interactions amplify the ecological consequences of MPs in the environment. Consequently, the ingestion of MPs embedded with contaminants can contribute to the transfer of toxic substances across terrestrial and aquatic food webs [46].

In freshwater ecosystems, MPs can adhere to plant tissues and subsequently transfer to herbivores that consume these plants. In a food chain, as MPs move from lower to higher trophic levels, they accumulate in animal tissues, leading to bioaccumulation. Because of this, freshwater organisms are often considered effective bio-indicators of MP contamination [47]. In a study performed by Raza et al. [48], it was seen that, MPs, particularly polyacrylamide, pose significant risks to aquatic environments by accumulating in fish tissues, disrupting antioxidant enzyme activity, and altering blood parameters. Their presence leads to oxidative stress, histological damage in vital organs, and overall impaired fish health, highlighting their toxic impact on aquatic ecosystems. NPs and MPs negatively impact marine organisms, but their toxicity toward marine bacteria remain less understood. In a study performed by Sun et al. [49], it was found that polystyrene NPs, more than MPs, inhibited the growth of *Halomonas alkaliphila*, disrupted ammonia conversion, and induced oxidative stress. These findings highlight the effect of plastic debris on marine microbial functions, potentially disrupting nitrogen cycles and ecological balance.

**Figure 3:**
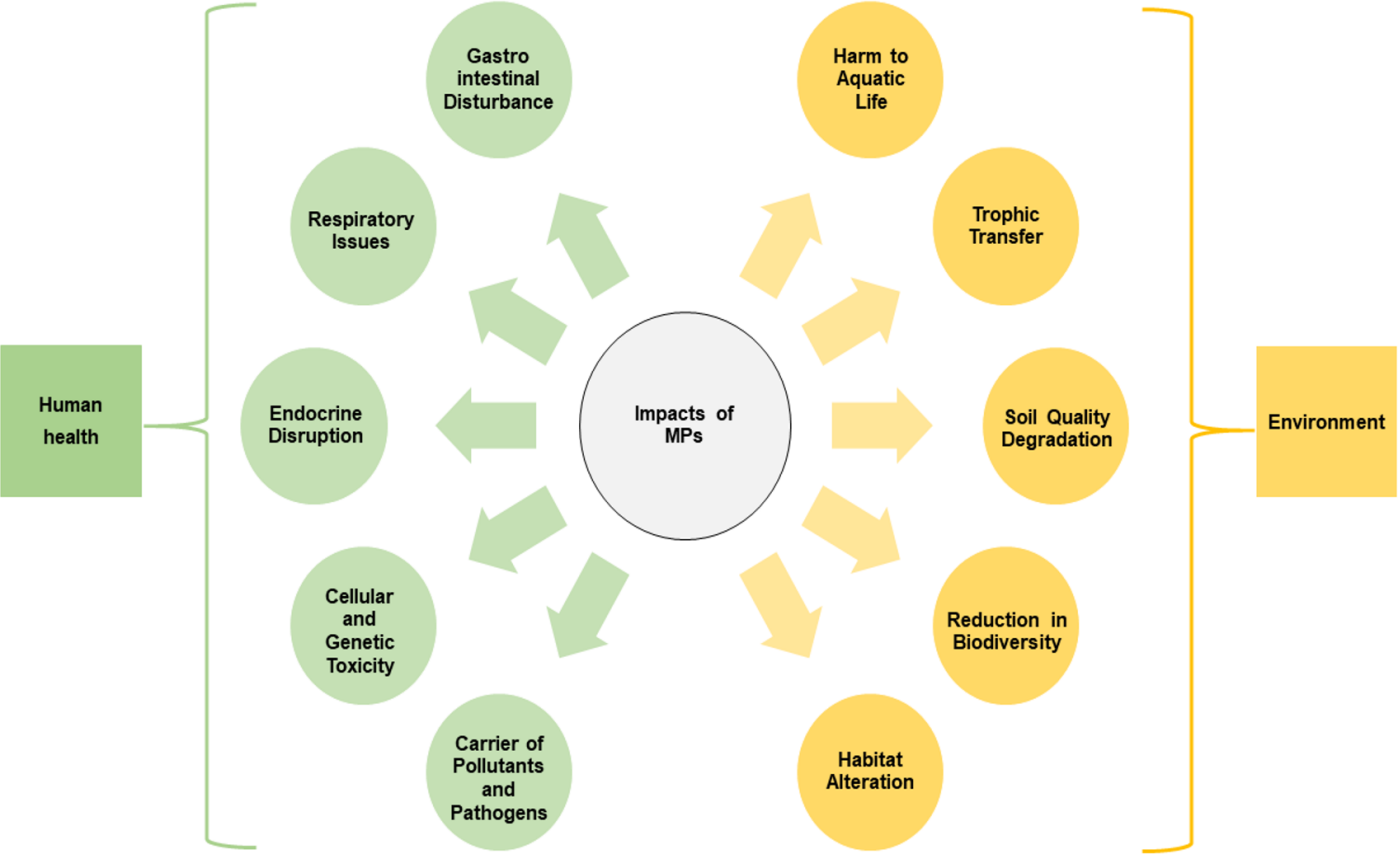
Impact of MPs on human health and environment.

When MPs enter the bodies of animals and humans through the food chain, they cause health problems including reduced birth rates, disruptions in the reproductive systems, altered sex ratios, and abnormal changes in body weight [45]. Understanding how MPs enter and affect the human body is essential, as their small size allows them to penetrate various biological barriers. MPs smaller than 5 μm can reach the alveoli, enter the circulatory system, and accumulate in the organs such as the brain, lungs, liver, spleen and digestive system. Those around 10 μm can breach cell membranes and the placental barrier, while MPs of 20 μm can reach internal organs. Exposure occurs through inhalation, posing risks to adults, while children face dangers from MPs in contaminated drinking water. Once inside the body, MPs can trigger neurotoxicity, cytotoxicity, oxidative stress, immune response, metabolic disruption and DNA damage [47]. According to a study conducted by Kumar et al. [50], humans are exposed to these MPs through various pathways, including seafood, water, agricultural products and beverages. MPs, along with toxic chemicals like polycyclic aromatic hydrocarbons and polychlorinated biphenyls, have been linked to reproductive, immune, and digestive systems through inhalation, ingestion, and dermal exposure. Polystyrene and polyvinyl chloride have been detected in human implants and are associated with carcinogenic effects. These plastics can induce oxidative stress, cytotoxicity, DNA damage, metabolic disruption, and immune responses, highlighting the urgent need for further research on their health impacts. Figure 3 shows various impacts of MPs on human health and environment.

### Removal of MPs from wastewater

**Figure 4:**
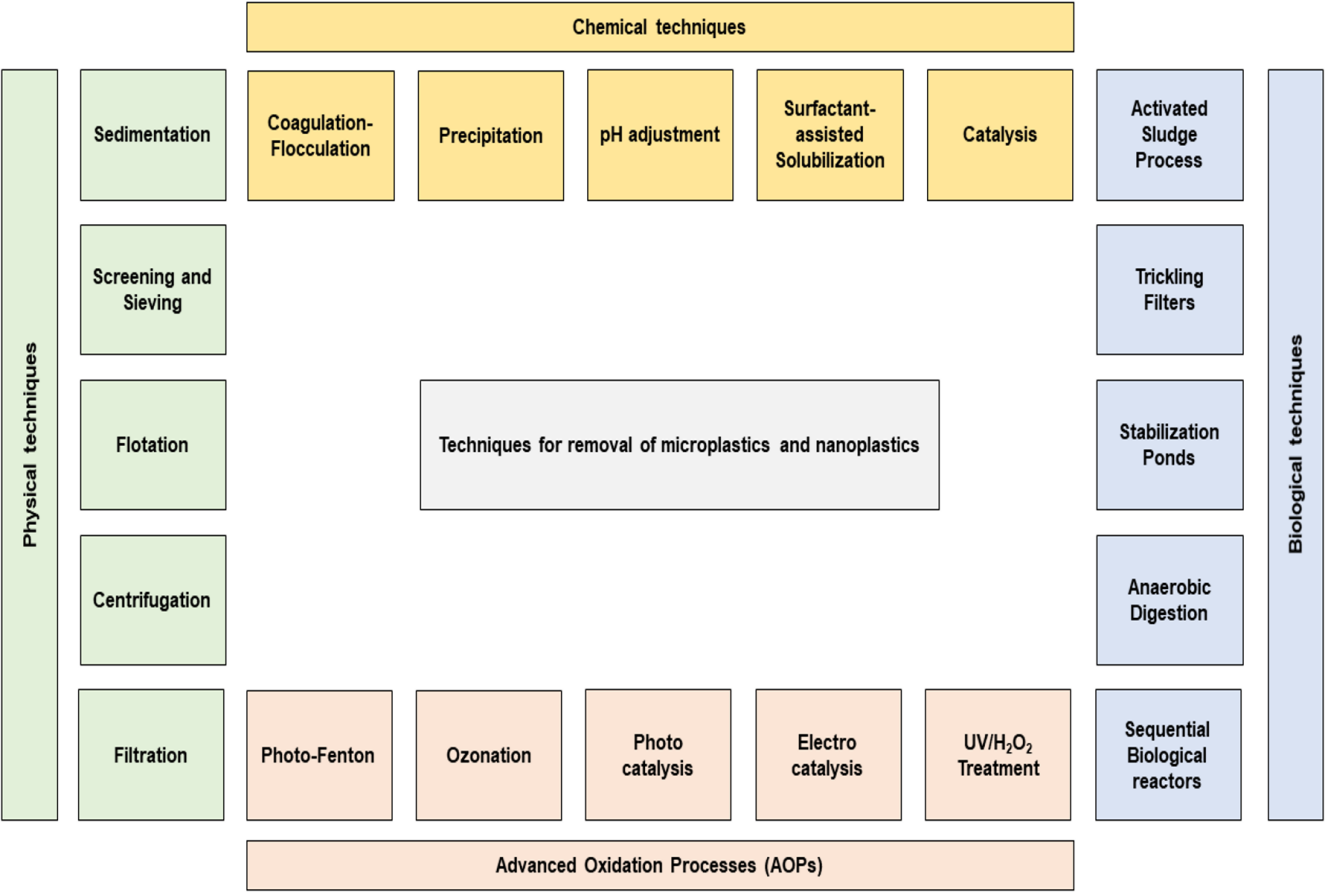
Common treatment methods for the removal of MPs from wastewater.

Figure 4 depicts various techniques for the removal of MPs from aqueous environments. Conventional approaches for removing MPs from water rely on a combination of physical, chemical and biological process [51]. Physical methods primarily facilitate the separation of larger plastic particles based on size and density [18]. Chemical processes are employed to destabilize and aggregate plastic particles, enhancing their removal through settling or filtration [52]. Biological treatments can entrap or partially degrade plastics via microbial activity, though typically with limited efficiency for persistent polymers. Advanced oxidation processes offer a more robust route for plastic degradation by generating highly reactive radicals through techniques such as Fenton and photo-Fenton reactions, UV/H_2_O_2_ systems, ozonation, TiO_2_, photocatalysis, and electrochemical oxidation [53]. These methods hold potential for breaking down MPs into smaller, less harmful by products, although optimization and scalability remain ongoing challenges.

### Physical processes

Various membrane filtration technologies have been employed to mitigate MP pollution, including microfiltration, membrane bioreactors, reverse osmosis, dynamic membranes, and ultrafiltration. Additionally, media filtration techniques, such as sand filtration and activated carbon particle filtration, have been utilized in drinking water treatment plants to enhance MP removal [54].

#### Chemical processes

Chemical treatment involves the use of specific reagents that initiate a series of chemical reactions aimed at enhancing the purification of water. This approach is particularly useful in removing MPs that are not effectively eliminated through physical or biological methods. During the chemical treatment, compounds are introduced to either break down MPs or facilitate their removal. These methods work by transforming MPs into less hazardous substances. Common chemical treatment techniques include coagulation, flocculation, precipitation, and electrocoagulation [18]. Even though both physical and chemical methods are found to be effective in the removal of MPs, they pose several challenges on the remediation of MPs, such as blocking pores and surfaces of membranes and increased amounts of coagulants [55].

#### Biological processes

Various microorganisms, including *Bacillus*, *Actinobacteria*, *Pseudomonas*, *Aspergillus*, *Penicillium*, *Cyanobacteria*, and different species of microalgae, have demonstrated the ability to degrade MPs such as low-density polyethylene (LDPE), high-density polyethylene (HDPE), PET, and polyester. These microorganisms have been identified in environments heavily contaminated with plastics, such as municipal landfills, dumpsites, polluted water bodies, and even in the digestive systems of plastic-feeding insects. Advances in biotechnology have further enabled the development of genetically modified organisms to enhance MP degradation. However, a significant concern with microbial biodegradation is the potential ecological impact of introducing these organisms into non-native environments, which may lead to unforeseen consequences. Additionally, some microorganisms produce toxic by-products during degradation, but this challenge can be addressed through the use of microbial consortia [56].

#### Advanced oxidation processes

Advanced oxidation processes (AOPs) represent a category of water treatment technologies that utilize reactive radical oxidation mechanisms. These processes generate reactive oxygen species (ROS) through methods such as photocatalysis, ozonation, and electrochemical activation, which effectively break down the polymer chains of MPs, leading to their mineralization. The primary AOP techniques used for removing MPs from water include ultraviolet-induced oxidation, ozone-based oxidation, photocatalysis (activated by UV, solar, or visible light), electrochemical oxidation, and persulfate-activated oxidation [57]. In photocatalysis, photons excite the catalyst, generating electron–hole pairs that trigger redox reactions with the pollutants adsorbed on its surface. In Fenton and Fenton-like processes, hydroxyl (•OH) radicals are formed when hydrogen peroxide reacts with a metallic active phase, such as Fe^2+^, facilitating the oxidation and breakdown of contaminants [58]. Table 2 depicts the advantages and limitations of various techniques for the removal of MPs.

**Table 2:** Techniques for removal of MPs and NPs from aqueous environments.

	Technique	Mechanism	Advantages	Limitations	Ref.

physical processes	sedimentation	allows particles to settle based on density difference between MPs and water	cost effective; suitable for large MPs	ineffective for smaller MPs and time consuming	[59]
	flotation	air bubbles adhere to MPs	quick operation, little space needs, adaptability of use, and moderate price	reagents for flotation, a hydrophobic surface, and entrainment of organic pollutants are all necessary	[18,60]
	centrifugation	MPs/NPs removed based by centrifugal force	rapid and efficient separation; scalable	energy-intensive; less effective for MPs with lower settling velocity	[61–62]
	filtration	MPs are physically trapped by means of a filter medium	adapted and customised to different scales; does not require chemical additives	clogging of filters and limited performance for very small MPs and NPs	[62]
	adsorption	materials with high affinity to MPs adsorb them to surfaces facilitating removal	high removal efficiency; can be regenerated and reused; removes other pollutants	low selectivity, prepared using an adsorbent	[63]

chemical processes	coagulation- flocculation	neutralizing charge of colloidal particles and subsequent removal through filtration	cost-effective; widely adopted	enormous amount of sludge generation	[18]
	electrocoagulation	electric current destabilizes and agglomerates microplastic particles	capable of removing other pollutants; scalable	electrode fouling less efficient for small particles	[64–65]

biological processes	activated sludge	MPs entrapped in microbial flocs followed by degradation and sludge formation	good removal efficiency	removal efficiency varies	[27,66]
	anaerobic–anoxic– aerobic activated sludge	removes MPs by a combination of anaerobic, anoxic, and aerobic zones, along with different sludge return strategies	cost effective; short hydraulic retention time	low removal rate; time consuming; generates substantial amount of sludge	[67–68]
	enzymatic degradation	Enzymes employed to degrade MPs	Efficient degradation of MPs	high cost; intricate process for enzyme development	[67,69]
	biodegradation	extracellular enzymes secreted by microbes depolymerise MPs	partial or complete degradation	low efficiency; time consuming	[54]
	membrane bioreactor	removal by perm-selective membrane along with biological process	easy integration with other processes; fine filter precision	poor removal of smaller MPs; high treatment cost	[18,54]

advanced oxidation processes	photo-Fenton	iron salts and H_2_O_2_ with UV generate hydroxyl radicals that degrade MPs	environment friendly and sustainable	requires UV and pH control; iron sludge formation	[70–71]
	ozonation	microplastic polymer can be split into functional groups that contain oxygen	low cost and high efficiency	high production cost; Environmental issues	[18,72]
	photocatalysis	generate ROS upon exposure to light	eco-friendly, long-lasting	requires a lot of energy (ultraviolet light)	
	electrochemical oxidation	electric current induced chemical reactions degrade MPs	high efficiency, degrading a number of organic contaminants, not requiring the addition of chemical agents, and not producing sludge	high cost of electrodes	[73]
	plasma treatment	plasma generates reactive species that chemically degrade MPs	have significant potential microplastic pollution	–	[62]

#### Nanoparticle-based removal

**Figure 5:**
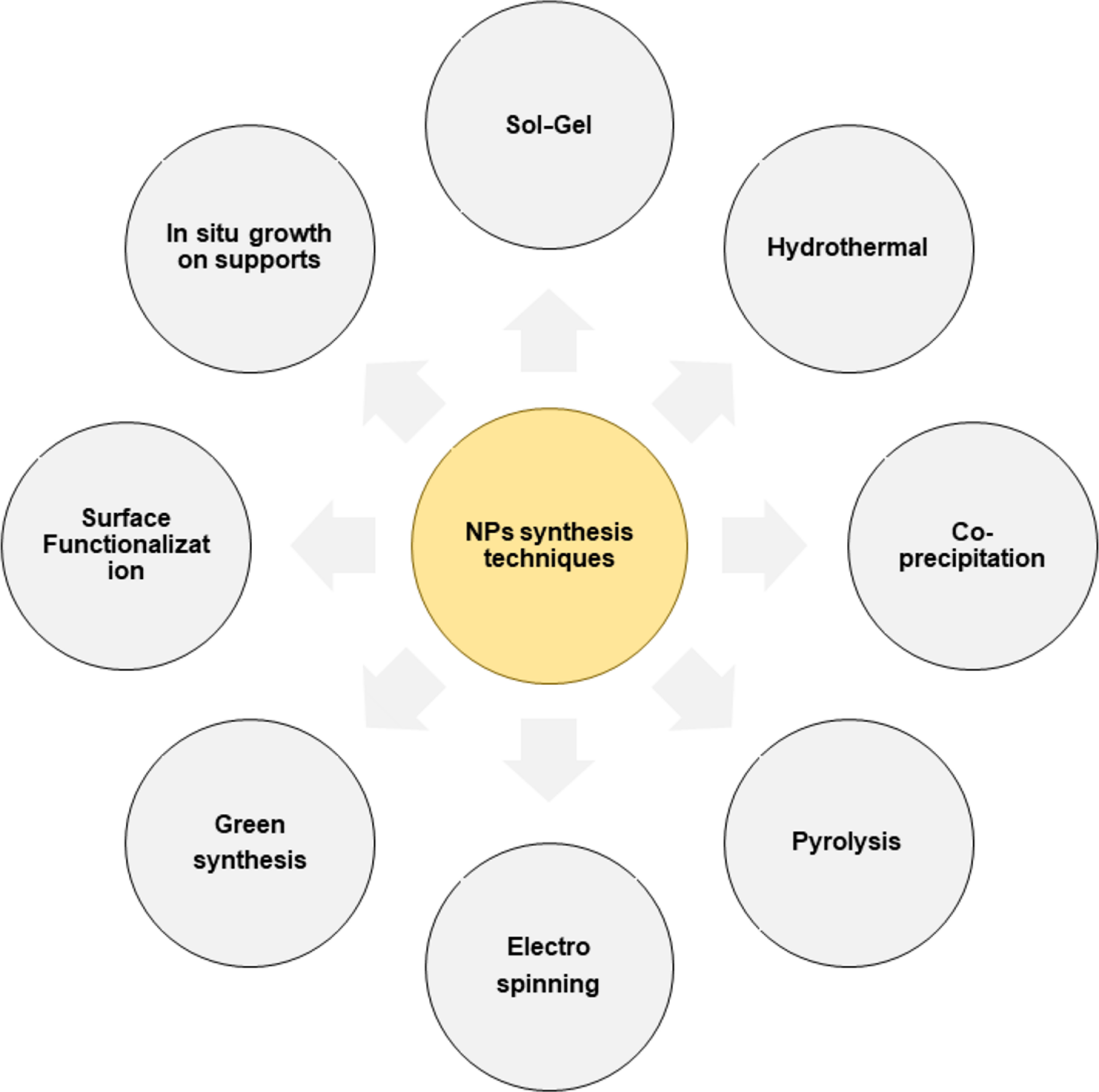
Different methods for synthesis of nanoparticles.

Advancements in characterization and synthesis techniques have enabled the manipulation of materials at the nanoscale, leading to innovations across various domains, including energy, electronics, and biomedical applications. Figure 5 depicts various techniques for synthesis of nanoparticles. Nanoparticle synthesis is essential for tailoring materials that effectively remove MPs. Various approaches allow researchers to customize structure and functionality based on application needs. For example, the sol–gel process transforms a colloidal solution into a gel, enabling precise compositional control and producing homogenous metal oxides suited for photocatalytic applications [74]. Hydrothermal synthesis uses high-temperature, high-pressure aqueous environments to yield well crystalized particles with controlled morphologies, while co-precipitation involves solvent displacement method, where acetone, ethanol, hexane are some of the solvents used [75]. In electrospinning, nanofibers are generated [76]. In parallel, green synthesis utilises biological entities such as plant extracts or microorganisms as reducing agents to produce eco-friendly nanoparticles, minimizing the used of hazardous chemicals [17]. Together, these techniques offer versatile strategies for developing nanomaterials that can be fine-tuned to optimize microplastic removal efficiency while adhering to sustainable practices.

**Figure 6:**
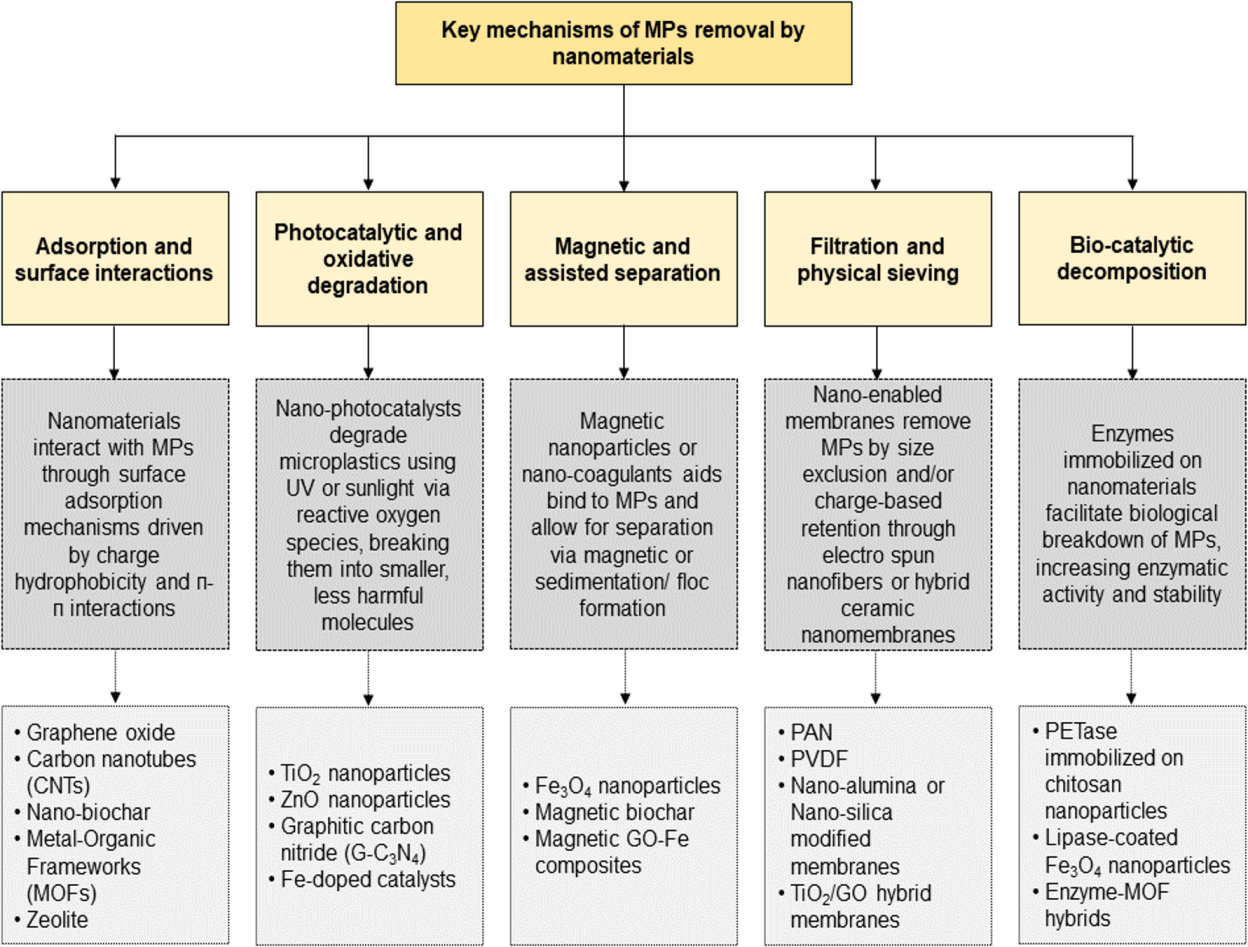
Key mechanisms of MP removal using nanoparticles.

In the field of water treatment, nanotechnology has been recognized for enhancing efficiency, affordability, effectiveness, and durability. These benefits stem from unique properties such as high specific surface area, increased reactivity, extensive functionalization, and size-dependent behaviour. By leveraging these characteristics, water treatment processes can be fine-tuned at the molecular or atomic level to selectively target specific contaminants [16]. Figure 6 illustrate the key mechanisms of MP removal using nanoparticles.

**Nanoparticles as adsorbent:** Adsorption is the most commonly used method for eliminating inorganic and organic pollutants from water and wastewater [77–81]. However, traditional adsorbents often exhibit limitations such as poor selectivity, low specific surface area, a limited number of active sites, and slow adsorption rate. In addition, regeneration cycles and short adsorption can affect the cost-effectiveness of the process. The removal of MPs by adsorbents primarily relies on hydrophobic interactions, electrostatic attraction and hydrogen bonding, which are influenced by their surface characteristics. Among various adsorbents, activated carbon and biochar have gained wide attention for treating water contaminated with MPs. Meanwhile, a higher cost of these adsorbents limit their use for the removal of MPs [82]. Research is actively exploring alternative adsorbents, with a particular focus on nanomaterial-based options. Among these, carbon nanotubes (CNTs), nanoscale metals, nanocomposites, and metal oxides offer a promising approach [55]. Nanoadsorbents possess a higher specific surface area and a large number of active sites, contributing to their rapid processing, enhanced selectivity, and extended adsorption–regeneration cycles [80]. In addition, nanomaterials also exhibit excellent reactivity, adaptability for functionalization and superior sorption capacity.

Carbon materials with graphene-like structures, made up of sp^2^-hybridized carbon atoms, have gained considerable interest for their use in water treatment technologies. Their abundance of functional groups, expansive surface area, and inherent hydrophobic nature make them highly effective in capturing various organic pollutants, including methylene blue, neutral red, and polycyclic aromatic hydrocarbons. Notably, the unique hexagonal honeycomb crystal structure imparts exceptional stability, allowing them to perform efficiently under challenging environmental conditions and across a wide pH range, making them reliable materials for pollutant removal [83]. Sun et al. [84] studied the removal of MPs from water using a sustainable adsorbent composed of graphene oxide and chitin. The elastic nature of the sponge retains its high porosity, enabling consistent and efficient adsorption across reuse cycles.

**Figure 7:**
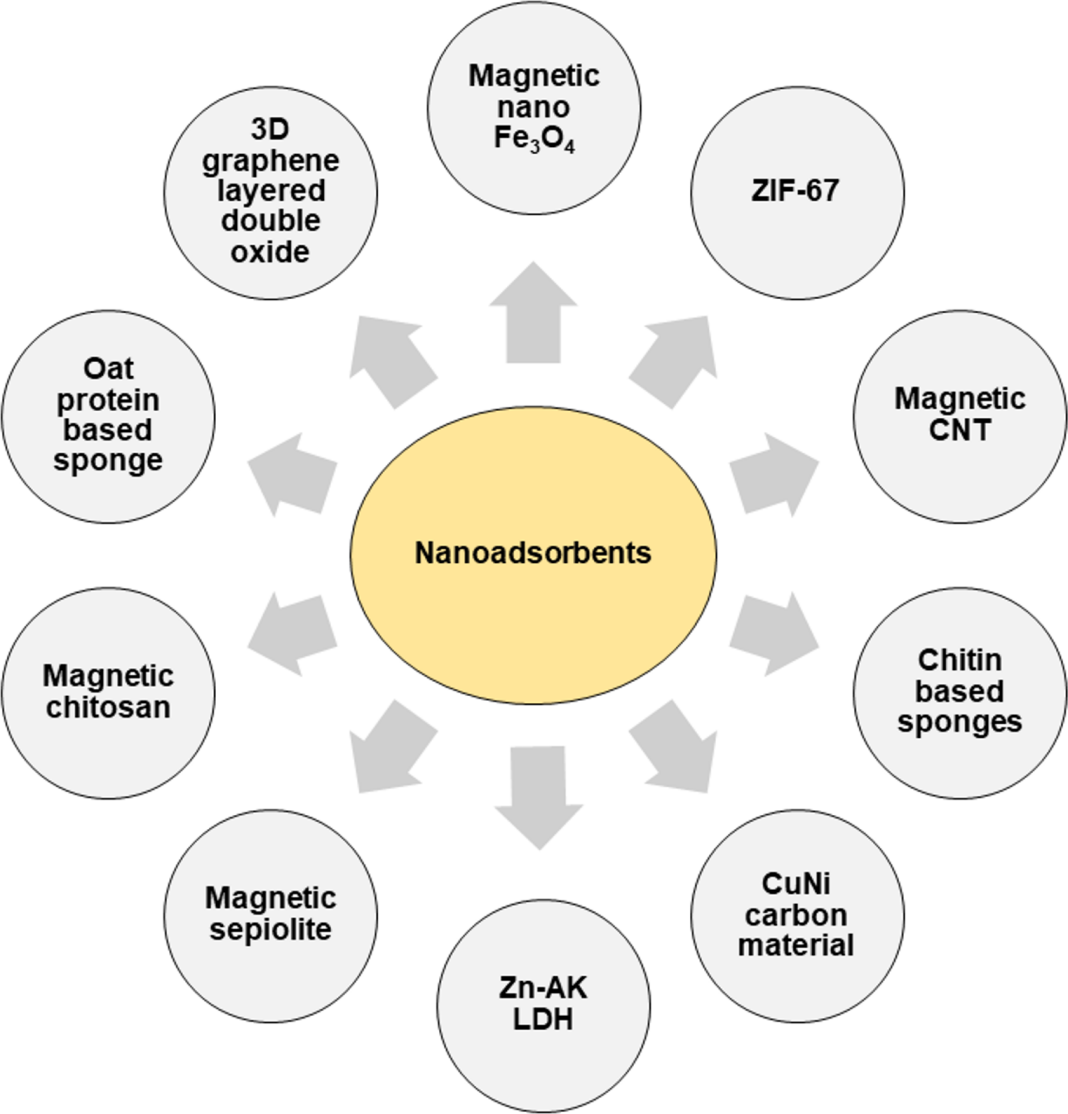
Different types of nanoadsorbents used for the treatment of MPs.

Layered double hydroxides (LDHs) are a distinctive group of 2D inorganic materials known for their remarkable physical and chemical characteristics. Their exceptional ion exchange ability, large surface area, high thermal stability, and customizable structural features have made them valuable in water purification process [85]. When subjected to heat, LDHs release interlayer water and anions. This results in the formation of double-layered oxides (LDO). Both LDO and LDHs have shown effectiveness in adsorption processes for removing organic pollutants, including MPs [86]. Figure 7 illustrate the various types of nanoadsorbents used for MP removal, while Table 3 present their operational conditions and corresponding removal efficiencies for different types of MPs.

**Table 3:** Nanoadsorbents for the removal of MPs and NPs.^a^

Nano adsorbent	MPs	Conc.	Adsorbent dosage	Sample pH	Contact time (min)	Max removal efficiency (%)	Kinetics	Isotherm	Ref.

magnetic nano-Fe_3_O_4_	PE, PP, PS, PET	0.5 g/L	1.3 g/L	–	150	>80%	–	–	[87]
ZIF-67	PS	5 mg/L	0.4 g/L	8	20	92.1	PFO	FI	[88]
magnetic CNT	PE, PET	5 g/L	5 g/L	–	300	100	–	–	[89]
chitin-based sponges	C-PS, A-PS and PS	1 mg/L	–	6–8	–	92.1%	–	–	[90]
CuNi carbon material	PS	10 mg/L	0.3 g/L	–	–	99.18%	PFO	LI	[91]
Zn-AK LDH	PS	250 mg/L	5 mg	4	120	100	GO	FI	[92]
magnetic sepiolite	PE, PP, PS, PET	10 g/L	0.8 g	–	10	98.4	–	–	[93]
polydopamine-enhanced magnetic chitosan	PET, PE, PS	300 mg/L	–	6–9	1440	97.3	PSO	FI	[94]
G@LDO	PS	100 mg/L	1 g/L	3–11	720	>80	PSO	LI	[83]
oat protein-based sponge	PS	1 mg/L	20 mg	4	–	85	PSO	LI	[95]

^a^PE – polyethylene, PS – polystyrene, PET – polyethylene terephthalate, C-PS – carboxylate modified polystyrene, A-PS – amine modified polystyrene, PFO – pseudo first order, GO – general order, PSO – pseudo second order, FI – Freundlich isotherm, LI – Langmuir isotherm, G@LDO – 3D graphene-like carbon assembled layered double oxide material.

**Nanoparticle-based photocatalysis:** Catalytic or photocatalytic oxidation, categorized under AOPs, is an effective method for eliminating trace pollutants and harmful microorganisms from water. Beyond this, photocatalysis serves as a valuable pretreatment technique, promoting the breakdown of persistent and toxic substances. It can also enhance the efficiency of subsequent chemical or biological treatments aimed at removing organic contaminants [96]. In photocatalysis, under UV illumination, electron–hole pairs are generated. These pairs interact with water molecules and dissolved oxygen to generate reactive oxygen species, such as hydroxyl radicals and superoxide anions. These highly reactive radicals act as strong oxidizing agents, attacking pollutants by breaking their chemical bonds, ultimately leading to their degradation and mineralization [97]. For a compound to undergo oxidation using a photocatalyst, its redox potential should be higher than the valence band edge of the semiconductor catalyst. In contrast, for reduction to occur, the redox potential must be lower than the conduction band edge. This is because, upon excitation, holes generated in the valence band participate in oxidation reactions, whereas the electrons excited to the conduction band drive the reduction processes [96].

The particle size of photocatalysts plays a very important role in the recombination of electrons and holes. Smaller particles exhibit higher photocatalytic activity due to their increased surface area, which allows for greater adsorption of pollutants and enhanced production of hydroxyl radicals. These benefits are particularly significant when the particle size is reduced to around 10 nm. Utilizing nanostructured semiconductors in photocatalytic applications proves to be more efficient, as a larger proportion of the photogenerated electron–hole pairs is available at the surface. Nanoparticles, owing to their high surface-to-volume ratio, demonstrate superior catalytic performance compared to bulk materials. Furthermore, the particle size of semiconductors influences their bandgap energy and crystalline structure, which in turn affects their redox potential and the spatial distribution of photo-induced charge carriers [98].

Tofa et al. [99] studied the photocatalytic activity of ZnO nanorods for breaking down LDPE, a common microplastic found in wastewater. Experimental results led to the proposal of a detailed degradation pathway. The process initiates when hydroxyl and superoxide radicals attack weak points in the polymer chains, typically structural flaws or chromophoric sites. This attack generates low-molecular-weight alkyl radicals within polyethylene. These reactive species then trigger various transformations, including chain cleavage, branching, crosslinking, and oxidation. Interaction with oxygen forms peroxy radicals, which extract hydrogen atoms from the polymer backbone, creating hydroperoxide intermediates. These intermediates break down into alkoxy radicals, which further react to produce carbonyl and vinyl groups, markers of advanced photo-oxidation. The presence of these functional groups in the treated polymer confirms successful degradation. The breakdown continues to yield volatile compounds such as ethane and formaldehyde, which can eventually be fully oxidized to carbon dioxide and water.

**Figure 8:**
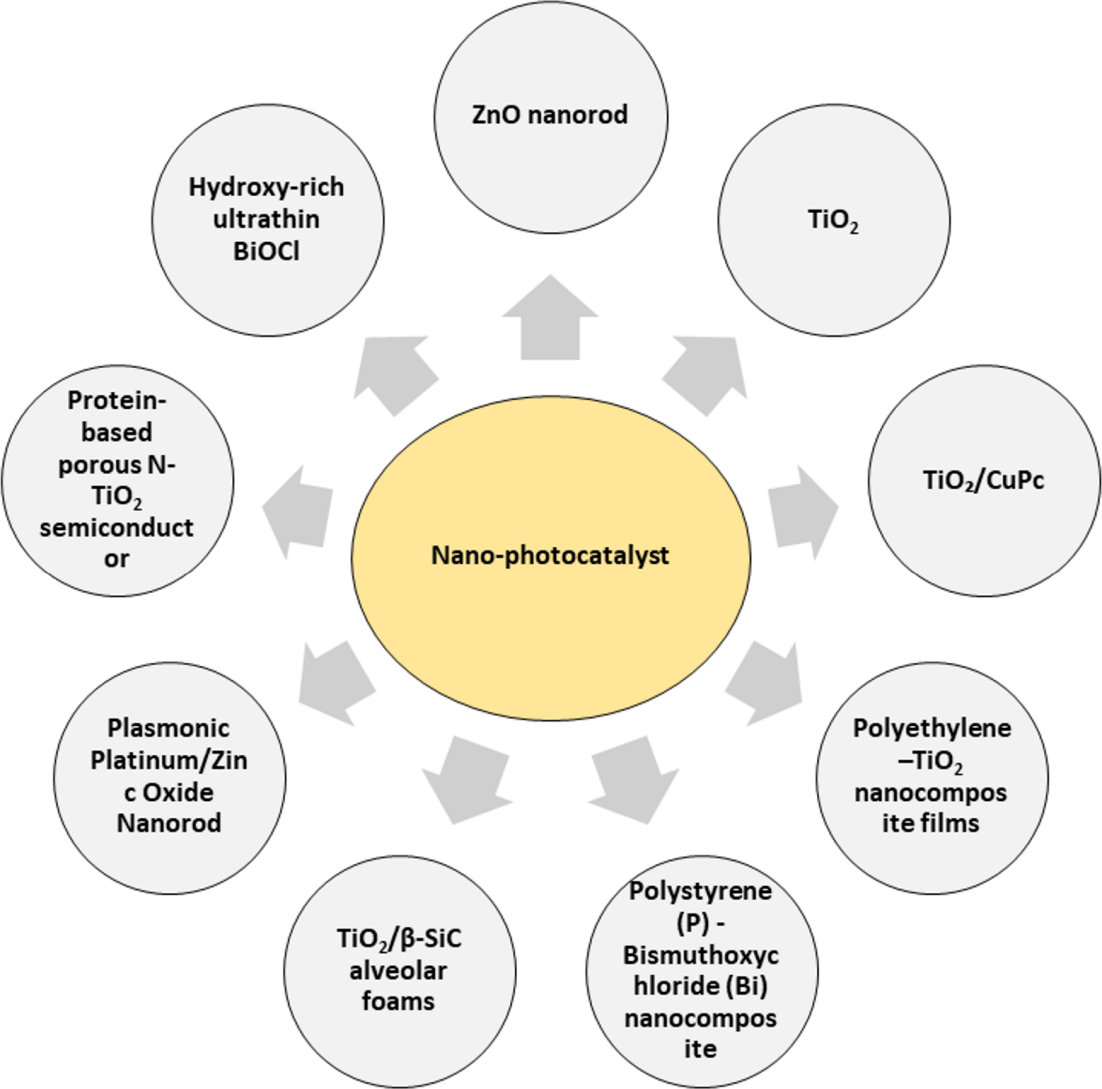
Different types of nano-photocatalysts used for the treatment of MPs.

Despite their advantages, using nanocatalysts in the form of dispersed powders poses several practical challenges, particularly concerning their recovery and reuse. These issues are critical, as there is a potential risk of nanoparticle release into the environment. To address these concerns, researchers have explored various strategies to immobilize the catalysts onto solid supports such as glass beads, fibres, silica, stainless steel, and textiles, or by embedding them within polymer matrices [86,100]. Figure 8 illustrates the various types of nanophotocatalysts used for MP removal, while Table 4 present their operational conditions and corresponding removal efficiencies for different types of MPs.

**Table 4:** Nano-photocatalysts used for MP removal.

Photocatalyst	MPs	Condition	Findings	Ref.

ZnO nanorods (ZnO NRs)	PP	Photocatalytic treatment was performed under visible light provided by a 120 W tungsten-halogen lamp (ES-HALOGEN), delivering an intensity of approximately 0.6 SUN (60 mW/cm^2^), as measured at 20 cm distance with a power meter.	65% reduction in average particle volume	[101]
TiO_2_ (Degussa P25)	PP	The photocatalytic degradation experiments were conducted inside a UV chamber equipped with a 75 Watt ultraviolet lamp, which emitted light primarily at a wavelength of 254 nm. During the tests, each sample was positioned 25 cm from the UV source and subjected to irradiation for durations of 100 and 500 hours. Throughout the exposure period, the chamber temperature was consistently maintained at 40 °C.	Degradation confirmed through increased carbonyl peak intensity and morphological changes	[102]
TiO_2_/CuPc (copper phthalocyanine-sensitized titanium dioxide)	PS	PS-TiO_2_ and PS-(TiO_2_/CuPc) films were irradiated under three 8 W fluorescent lamps (310–750 nm), total light intensity 1.75 mW/cm^2^ (UV portion ≈0.05 mW/cm^2^), at 7 cm distance. The setup was in ambient air at ≈298 K in a sealed lamp box (30 × 25 × 15 cm).	Enhanced degradation efficiency compared to pure TiO_2_: higher PS weight loss rate, lower average molecular weight, reduced volatile organic compounds, and increased CO_2_ production. ROS generated on the catalyst surface play a crucial role in chain scission	[103]
titanium dioxide (TiO_2_)	HDPE	HDPE was blended with 3 wt % TiO_2_ using a torque rheogoniometer at 150 °C, 60 rpm for 10 min. UV irradiation was performed using a xenon lamp (0.51 W/m^2^ at 340 nm, 65 °C, 25 cm distance) for up to 400 hours	The HDPE/TiO_2_ composites maintained high solar reflectance after UV irradiation. The solar reflectance after UV exposure depended on changes in crystallinity, surface roughness, and the structure of TiO_2_. Anatase TiO_2_ particles slightly increased solar reflectance in the near-infrared region post-UV irradiation, suggesting a potential approach to achieve high reflectance in polymeric materials. Temperature tests confirmed that the cooling performance correlated with solar reflectance measurements.	[104]
TiO_2_ nanoparticles	PEF	The UV-induced degradation of polyethylene (PE)–TiO_2_ composite films was conducted in a custom-built chamber equipped with two 15 W ultraviolet lamps emitting at 365 nm. The films were placed 20 cm from the light source inside a lamp housing box (dimensions: 55 cm × 35 cm × 30 cm), and continuously exposed to UV light for a duration of 300 hours	TiO_2_ showed better photocatalytic property under UV radiation which showed weight loss up to 18% in 300 h compared to commercially available TiO_2_	[105]
polyethylene–TiO_2_ nanocomposite films	PEF	The solar degradation experiments were conducted by placing three sets of film samples in petri dishes and exposing them to natural sunlight during summer months, daily from 9:00 a.m. to 4:00 p.m., under light intensities ranging between 75,000 and 95,000 lux.	When exposed to solar radiation, the composite films showed a significant weight loss of 68% within a period of 200 h	[106]
novel photodegradable low-density polyethylene‒TiO_2_ nanocomposite film	LDPE	The films were placed in quartz vessels and were irradiated under a 30 W ultraviolet lamp with a primary wavelength of 254 nm. Each sample was weighed every 48 h by an accurate balance	After 400 hours of UV light exposure, the composite film exhibited a weight reduction of 68.38%, along with significant declines in molecular parameters – showing a 94.56% decrease in the weight-average molecular weight (*M*_w_) and a 93.75% decrease in the number-average molecular weight (*M*_n_).	[107]
polystyrene (P) - bismuthoxychloride (Bi) nanocomposite films	PS	The photo-oxidation reaction of neat PS and P/Bi composite samples performed under 500 W Halogen luminaire lamp, where the temperature during irradiation was maintained around 298 K. The light intensity is measured to be 15 × 103 lx at 5 cm away from the lamp. Size of the film sample was about 78 cm^2^.	The outcomes acquired the surfaces of as-prepared polymer films with BiOCl were effectively degraded by photooxidation reactions.	[108]
TiO_2_/β‑SiC alveolar foams	PMA and PS NPs	Three TiO_2_–P25/β-SiC foam monoliths were placed inside a tubular quartz reactor. Each foam sample (10 g) contains 10 ± 1 wt % of TiO_2_. The reactor was externally surrounded by four UV-A lamps (Philips T5 15W/10 Actinic BL, λ_max_ = 354 nm), positioned at a 1 cm distance from the quartz wall. Each lamp emitted approximately 4.5 mW/cm^2^ at the reactor surface.	about 50% of the carbon of polymethylmethacrylate nanobeads is degraded in 7 h	[108]
plasmonic platinum/zinc oxide nanorod	LDPE	The photocatalytic degradation studies of ZnO and ZnO-Pt substrates were carried out using a 50 W dichroic halogen lamp in ambient air, providing visible light illumination with an intensity of approximately 60–70 × 10^3^ lx at a distance of 10 cm from the samples. The photocatalysis of low-density polyethylene film samples (size: 1 cm × 1 cm) was performed for 175 hours in a Petri dish containing the synthesized catalysts and deionized water under the same illumination conditions.	The plasmon-enhanced ZnO-Pt photocatalyst demonstrated effective degradation of microplastic contaminants, specifically residual low-density polyethylene (LDPE) films in water. Compared to unmodified ZnO nano rods, the ZnO-Pt composite exhibited approximately 13% greater efficiency in facilitating the oxidative degradation of LDPE films.	[109]
protein-based porous N–TiO_2_ semiconductor	HDPE	Photocatalytic experiments using the control sample, sol–gel synthesized N–TiO_2_, were conducted by introducing 200 mg of isolated MPs along with 100 mL of distilled water into a batch reactor coated with N–TiO_2_. All photocatalytic reactions were carried out within a sealed chamber at ambient temperature. The system was exposed to visible light emitted by a 27 W fluorescent lamp, positioned 120 mm away from the samples, for a continuous duration of 20 hours.	Photocatalytic degradation of the HDPE/N-TiO_2_ composite in water at room temperature showed no measurable mass loss in the absence of light exposure. However, under visible light irradiation, the system exhibited progressive degradation for the initial 18 hours, after which the reaction plateaued. The total mass loss was determined to be 6.40%, with a reaction rate approximately three times higher than that observed in the solid-state photocatalytic setup.	[110]
hydroxy-rich ultrathin BiOCl	PE	Microplastic degradation experiments were performed using a circulating water system illuminated by a 250 W xenon lamp. In each test, 1 g/L of micron-sized polyethylene (PE-S) or 10 g/L of millimeter-scale plastics such as HDPE, PP-W, PP-B, PP-R, PA66, or POM was suspended in 100 mL of water. Subsequently, photocatalyst was introduced at a concentration of 1 g/L. Following a 5 hour irradiation period, the lamp was switched off, and the reaction mixture was filtered using a 300 mesh stainless steel screen to separate the plastic residues from the solution.	BiOCl-X exhibited significantly enhanced photocatalytic performance in microplastic degradation, with the resulting mass loss being 24 times greater than that achieved using conventional BiOCl nanosheets.	[111]

^a^PE – polyethylene, PS – polystyrene, HDPE – high density polyethylene, LDPE – low density polyethylene, PMA – polymethylmethacrylate, PS NPs – polystyrene nanoplastics, PEF – polyethylene film, POM – polyoxymethylene.

**Figure 9:**
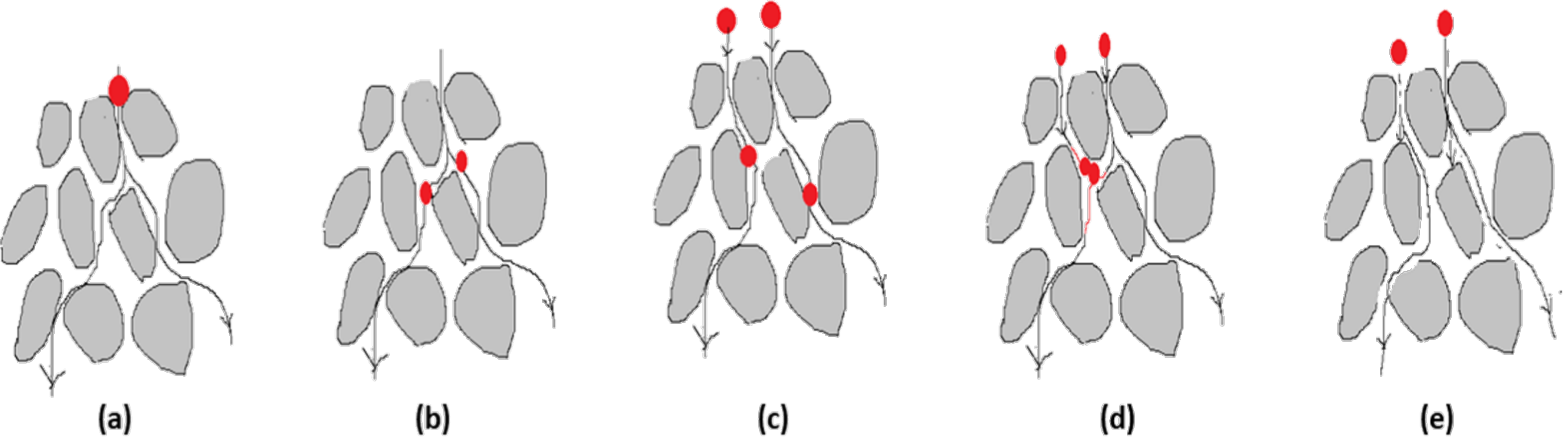
Physical mechanism of MP removal (a) mechanical straining, (b) sedimentation, (c) interception, (d) flocculation, and (e) impaction.

**Nanoparticle-based membranes and membrane processes:** Membranes function as selective barriers that permit the passage of specific substances, while blocking others. Based on pore size, membranes are generally classified into four main categories: ultrafiltration (UF), microfiltration (MF), reverse osmosis (RO), and nanofiltration (NF). MF membranes typically remove particles between 0.08 and 2 μm, UF targets those in the 0.005–0.02 μm range, and NF membranes capture particles around 0.002 μm. RO membranes, in contrast, are widely applied in desalination processes for treating seawater and brackish water [111]. The physical mechanisms involved in the removal of pollutants are schematically represented in Figure 9 and explained in Table 5 [112].

**Table 5:** Physical mechanism of pollutant removal.

Mechanism	Description

mechanical screening	MPs larger than membrane pores are physically blocked
sedimentation	heavier plastic particles settle near the membrane surface or within membrane modules due to gravity or flow velocity changes
flocculation	coagulated or bio-aggregated MPs clump together and form larger aggregates, making them easier to retain
interception	particles following water flow lines come into contact with membrane surface and are trapped, even if smaller than pore size
impaction	MPs deviate from water streamlines due to inertia and collide with the membrane surface or embedded nanoparticles

Membrane technology faces a key limitation in balancing selectivity with permeability, often resulting in a compromise between the two. High energy demands further restrict the widespread adoption of pressure-driven membrane systems. Additionally, membrane fouling not only raises energy consumption but also adds complexity to system operation and design. This fouling also shortens the operational lifespan of membranes and their associated modules. The efficiency of a membrane system is primarily governed by the material used in its construction. Enhancing membranes with functional nanomaterials provides a promising path to boost permeability, reduce fouling, and increase both mechanical and thermal durability, while also enabling advanced capabilities such as self-cleaning and pollutant breakdown [113]. Recently, the utilization of nanomaterials has gained significant attention for creating next-generation membranes with enhanced anti-fouling and anti-scaling, and improved transport capabilities. Among the most frequently used nanomaterials in membrane fabrication are zeolites, various metals and metal oxides, as well as carbon-based materials such as CNTs and graphene derivatives [86]. The nanomaterials that can be used membrane components for the removal of MPs are discussed in detail below.

**Figure 10:**
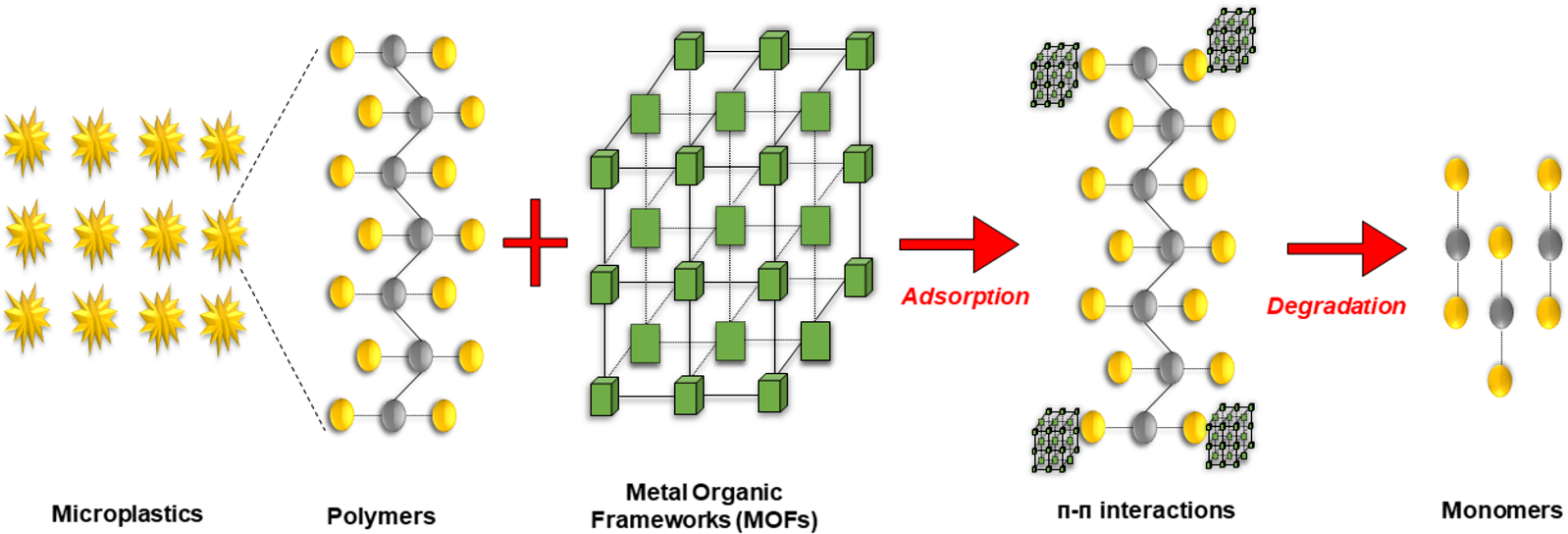
Mechanism of MP removal using MOF.

*Metal-organic frameworks*: Metal-organic frameworks (MOFs) are a complex and relatively new category of highly porous nanomaterials that have gained attention over the past two decades, showing broad potential in wastewater treatment. These crystalline materials are formed through coordination bonds that connect clusters or metal ions with multidentate or bidentate organic ligands. Sometimes MOFs are referred to as porous coordination polymers. MOFs are created by combining clusters or metallic ions with inorganic or organic ligands. The metallic component consists of metal ions or clusters with organic or inorganic ligands like sulfonate, phosphonate, carboxylates, and heterocyclic compounds [114]. MOFs possess a combination of properties including as low density, large porous structure, and high adsorption capacity, which makes them suitable for wastewater treatment [115]. In addition, the strong coordination bonding within their framework results in a large specific surface area, which in turn offers the potential to control membrane pore size and enhance the removal efficiency of nanoparticles and MPs. Membranes developed using MOF-based nanomaterials offer scalable control over surface charge and pore size, enabling high separation efficiency along with favourable thermodynamic properties. Although extensive studies have been conducted regarding MOF-modified membranes for the removal of salts and organic dyes, their application in microplastic removal remains largely unexplored. Because of their excellent compatibility, highly porous structure, and adjustable pore dimensions, designing a durable and compact MOF nanocomposite membrane could provide an effective strategy for MP filtration [116]. The mechanism involved in the removal of MPs using MOFs is represented in Figure 10.

Gnanasekaran et al. [117] developed a novel composite membrane by integrating sustainably synthesized MIL-100 (Fe) MOF nanoparticles into a polysulfone (PSF) matrix. This PSF/MIL-100 (Fe) membrane exhibited substantial improvements over the unmodified PSF membrane (M0), particularly in terms of membrane structure, surface hydrophilicity (with the water contact angle decreasing from 84.6° ± 1° to 64.2° ± 1.2°), work of adhesion, wetting energy, porosity, and pore dimensions. Among various loadings, the membrane with 0.5 wt % MIL-100 (Fe) (M0.5) showed the best overall performance. It delivered a pure water flux over ten times higher than M0 and achieved over 99% removal efficiency for methylene blue (MB). Additionally, M0.5 demonstrated excellent resistance to fouling when filtering MB dye and microplastic mixtures. The membrane maintained its high performance across different MB concentrations, MPs levels, and transmembrane pressures. The study concludes that PSF/MIL-100 (Fe) composite membrane offers a promising solution for the removal of MPs [117].

Chen et al. [118] developed a series of zirconium-based MOF foam materials with interconnected pores, excellent stability, and high structural uniformity for the removal of MPs from both seawater and freshwater. These materials proved effective across a wide range of MP concentrations and types. Among them, UiO-66-OH@MF-3 demonstrated the highest efficiency, achieving MP removal rates of up to 95.5% ± 1.2%, while maintaining consistent performance across multiple reuse cycles and large-scale filtration tests. Additionally, a lab-scale automatic filtration system powered by solar energy was designed and implemented, showcasing the feasibility of integrating these high-performance foam materials into sustainable filtration technologies. This combined approach offers promising potential for advancing innovative solutions in MPs remediation.

You et al. [119] synthesized a robust composite material, ZIF-8@Aerogel, by growing Zn-based MOF ZIF-8 directly onto wood-derived aerogel fibres. This composite demonstrated effective MP removal in both freshwater and seawater simulations. Specifically, it achieved a removal of 85.8% for polystyrene particles (90–140 nm) and 91.4% for poly(1,1-difluoroethylene) particles (60–110 nm). These findings highlight the potential of this material as a promising approach for eliminating small scale MPs from environmental water sources.

*MXenes and metal oxides*: MXenes, two-dimensional nanomaterials, have received interest across scientific fields owing to their exceptional chemical and thermal stability. Their chemical formula is M*_n_*_+1_X*_n_*T*_x_* (*n* = 1, 2, or 3), where M denotes an early transition metal such as titanium, molybdenum, or vanadium, while X denotes carbon and nitrogen, and T*_x_* represents surface functional groups like oxygen, fluorine, hydroxy, or hydrogen. MXenes are synthesized by selective removal of A-layers with elements from group IIIA or IVA, such as aluminium or silicon, from a parent material. This process results in a nanomaterial with an expansive surface area and layered, folded structures. These 2D materials are known for their chemical robustness, surface functionality, hydrophilicity, excellent electrical conductivity, and eco-friendly nature. Due to these properties, MXenes, particularly titanium carbide derivatives (Ti_3_C_2_T*_x_*), have become widely explored for applications such as water treatment and purification [114].

Yang et al. [120] introduced for the first time a membrane with an enhanced water permeability. This membrane was obtained by selectively etching Co_3_O_4_ nanoparticles embedded within Ti_3_C_2_T*_x_* nanosheets, followed by vacuum filtration. The resulting h-Ti_3_C_2_T*_x_* nanosheets possess a porous, flat structure with 25 nm diameter holes ideal for MP separation. When tested with fluorescent polystyrene (FP) microspheres of varying sizes as MPs models, the membranes demonstrated exceptional removal efficiency of up to 99.3%. Additionally, a high water flux of 196.7 L·h^−1^·m^−2^·kPa^−1^ was recorded, surpassing or matching the performance of most membranes fabricated from unmodified two-dimensional nanomaterials. The material exhibits physicochemical stability, excellent permeability, and superior MP removal capabilities; hence, h-Ti_3_C_2_T*_x_* membranes show strong promise for practical use in filtering MPs and other suspended particles from wastewater.

Urso et al. [121] introduced a novel method for real-time capture and detection of NPs in a three-dimensional environment using multifunctional microrobots derived from MXene-based oxides. The fabrication process involves thermally transforming Ti_3_C_2_T*_x_* MXene into multilayered TiO_2_ with photocatalytic properties, followed by coating with platinum and decorating the surface with magnetic γ-Fe_2_O_3_ nanoparticles. These engineered γ-Fe_2_O_3_/Pt/TiO_2_ microrobots exhibit light-driven, fuel-free movement with six degrees of freedom due to their negative photogravitaxis. Their self-propelling capability, combined with tunable surface charge (zeta potential), enables rapid attraction and capture of nanoplastics onto their surfaces and between layered structures. The magnetic nature of the microrobots allows for easy retrieval. Acting as mobile platforms for preconcentration, these microrobots facilitate the electrochemical sensing of nanoplastics using inexpensive, portable electrodes. This proof-of-concept offers promising potential for real-time, on-site detection and subsequent removal of nanoplastics from aquatic systems.

**Figure 11:**
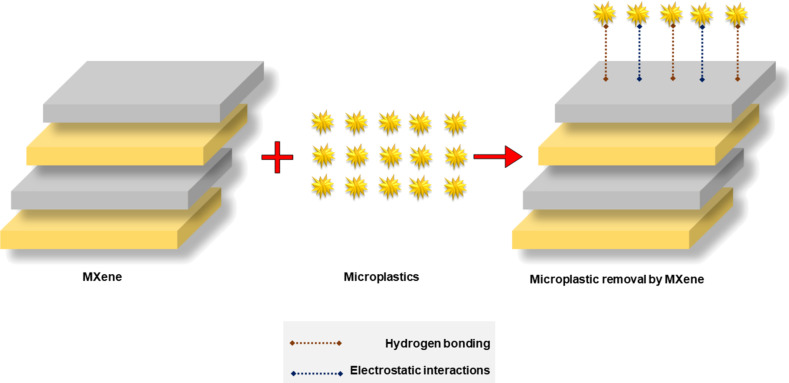
Removal mechanism of MPs using MXene.

MXenes have emerged as promising materials for antibacterial applications and have shown strong potential for the use in water purification membranes due to their excellent film-forming ability and enhanced mechanical strength. These properties also support their application in creating selective membranes for water desalination. Despite these advantages, the use of MXenes in certain areas remain limited. Their ultrathin 2D structure can present challenges, including unpredictable pore distribution and a tendency to collapse [122]. As a result, further research is necessary to fully explore and optimize MXenes’ capabilities, particularly for the removal of MPs from water. The mechanism involved in the removal of MPs using MXenes is shown in Figure 11.

To develop lasting strategies for combating water pollution, integrating metals and metal oxides into membrane systems has emerged as a promising approach. Materials such as silver, ZnO, TiO_2_, and iron oxide are frequently used in membrane construction due to their high reactivity and effective catalytic behaviour. These membranes are not only simple to engineer but also exhibit strong antibacterial activity. Additionally, their ability to trigger photocatalytic reactions under UV light makes them effective for breaking down MPs. Among these materials, silver is especially valued for its suitability in creating corrosion-resistant surfaces. Membranes composed of metals and metal oxides exhibit strong adsorption capabilities due to electrostatic interactions, making them highly effective in capturing MPs. Their fast adsorption kinetics support rapid water purification, and surface modifications can further enhance their performance and selectivity. These membranes are also versatile and applicable in various treatment scenarios. However, potential drawbacks include the leaching of toxic metal ions or nanoparticles, which may pose environmental and health concerns. Over time, fouling and clogging caused by MPs and other pollutants can reduce their operational efficiency, necessitating regular cleaning. Moreover, high production costs limit their practicality for widespread use in large-scale water treatment facilities [114].

**Figure 12:**
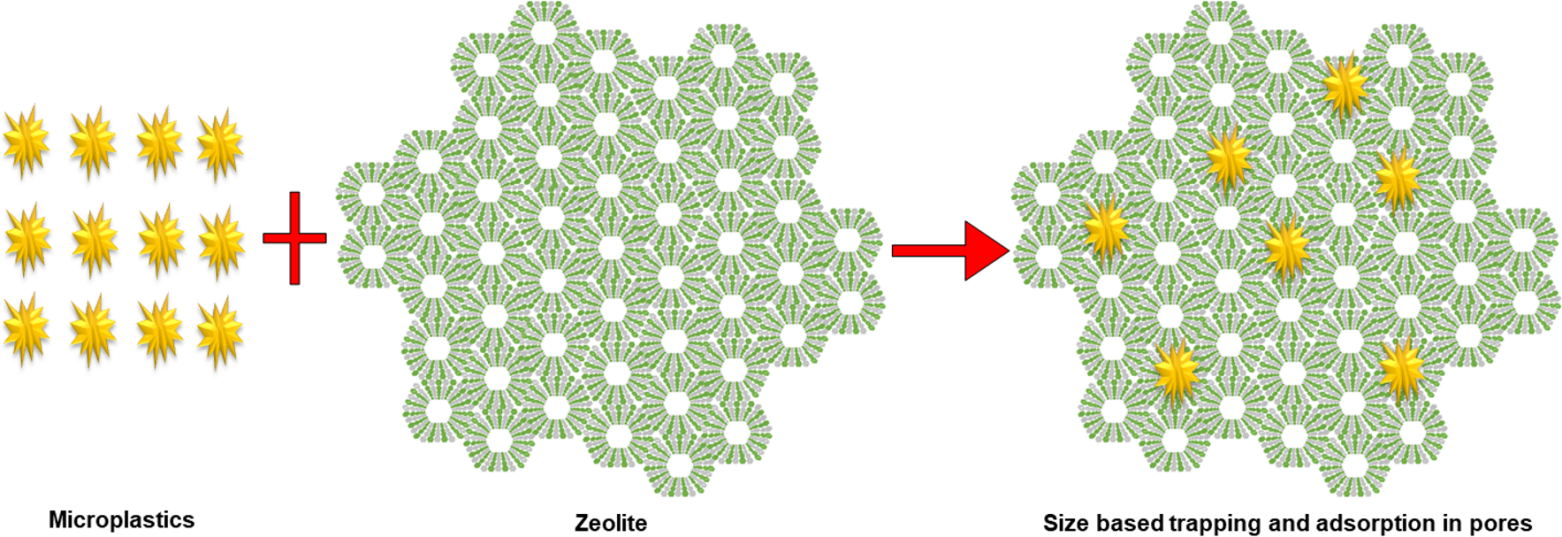
Removal mechanism of MPs using zeolite.

*Zeolite and carbon nanomaterials*: Research has shown that zeolite membranes can retain both their adsorption capability and structural stability across a range of environmental conditions, making them suitable for long-term applications in water treatment. Although zeolites have not yet been widely employed in membrane form specifically for microplastic removal, their highly porous structure provides a strong ability to capture MPs from water. Their design can be tailored to selectively adsorb different types of MPs depending on factors such as charge, size, and chemical composition. Being naturally abundant and environmentally friendly, zeolites offer a cost-effective option for sustainable water purification. However, challenges such as cost and consistent supply of high-quality zeolites can impact their large-scale application. Additionally, their adsorption performance can decline under extreme pH conditions, necessitating pH adjustment to achieve optimal efficiency [114]. The mechanism involved in the removal of MPs using zeolite is shown in Figure 12.

Membranes enhanced with carbon-based nanomaterials present several benefits for removing MPs from water. Their exceptionally large surface area and distinct structural features contribute to effective microplastic capture. Materials like CNTs also offer excellent mechanical strength and can be engineered for durability under extreme conditions, making them reusable and cost-effective in the long run. However, the high production cost of carbon nanomaterials may hinder their adoption in large-scale water treatment systems. Environmental concerns also arise due to the potential release of these nanoparticles, which may pose risks to both human health and ecosystems. Additionally, carbon nanomaterials often aggregate in water, diminishing their adsorption efficiency, and their fabrication typically involves advanced techniques and costly equipment, which further limits widespread use [114]. The mechanism involved in the removal of MPs using each material is discussed in Table 6.

**Table 6:** Removal mechanism of MPs using advanced material.

Material	Removal mechanism	Examples	Reactions	Ref.

metal-organic frame-works (MOFs)	* adsorption via π–π interactions between MOF ligands and plastic surfaces (e.g.: polystyrene) * electrostatic interactions with charged nanoplastics * porous structure enables size-effective trapping and high surface area adsorption	* ZIF-8 (possess positive charge) was found to remove MPs via electrostatic attraction * through phase separation MS@ZnCo-ZIF@HDTMS sponges are used as absorber matter for MPs removal	no degradation reaction, but adsorption occurs via π–π and Coulombic interactions	[123]

MXenes and metal oxides	* electrostatic interaction between negatively charged MXene surfaces and positively charged MPs * hydrophobic/hydrophilic interactions depending on surface terminations * metal oxides (like TiO_2_, Fe_3_O_4_) can also enable photocatalytic degradation under sunlight	* the hydrophilic nature of Ti₃C₂T_x_ (h-Ti₃C₂T_x_) nanosheet membranes demonstrated high efficiency in removing microplastics from wastewater * the integration of silver (Ag) metal with TiO₂ nanowires significantly improved the elimination of polypropylene microplastics.	photocatalysis: TiO_2_ + *h*ν → TiO_2_ (e^−^ + h^+^) h^+^ + H_2_O → •OH •OH + polymer → CO_2_ + H_2_O	[120,124]

zeolites	* ion exchange and adsorption due to high cation exchange capacity * surface electrostatic interaction enhances selective binding of charged MPs	* magnetically activated biochar-zeolite composite demonstrated a higher adsorption capacity for the removal of polystyrene microplastic	no degradation. Relies on surface polarity and ion-exchange capability	[125]

carbon-based nanomaterials	* includes graphene, carbon nanotubes, and activated carbon * hydrophobic interactions dominate between carbon surface and polymer chains * high surface area promotes multilayer adsorption	* CNTs exhibited an adsorption capacity of 1100 mg/g for polyamide, 1400 mg/g for PET, and 1600 mg/g for PE.	adsorption only	[126]

### Challenges and advancements in MP detection

The elimination of MPs from water bodies is a key challenge in controlling environmental pollution. Although various technologies have been designed to address this issue, most are still undergoing experimental validation in laboratory settings. Some studies have also explored the potential of wastewater and drinking water treatment facilities in removing MPs. Meanwhile, because of the vast volume of water processed daily, a considerable quantity of MPs may escape into the natural ecosystems through treated water discharge [54].

Advanced strategies for MP mitigation demonstrate diverse mechanisms and operational advantages. Hybrid systems combining ceramic ultrafiltration membranes with photocatalytic reactors offer high removal efficiency (up to 99.9%) and reduced membrane fouling, but are limited by high costs and system complexity. Preventive approaches, such as glutenin-genipin cross-linked coatings, effectively reduce plastic shedding under harsh conditions using biodegradable materials; however, they address only source control and lack proven scalability. Imine-functionalized mesoporous magnetic silica nanoparticles, enhanced through machine learning (ML) optimization, enable dual removal of MPs and organic pollutants with efficient magnetic recovery. Despite their potential, concerns remain regarding synthesis complexity, environmental safety of nanomaterials, and real-water applicability. These emerging methods reflect promising directions but require further validation for widespread adoption [127]. Rushdi et al. [128] developed imine-functionalized mesoporous magnetic silica nanoparticles for the simultaneous removal of polystyrene MPs and organic pollutants. The nanoparticles exhibited strong magnetic recovery and high adsorption selectivity due to imine surface groups. Machine learning was applied to optimize operational parameters, enhancing removal efficiency while minimizing sorbent use. The system demonstrated stable performance across multiple reuse cycles, highlighting its potential as a smart, dual-function remediation material [128].

Green materials are increasingly being explored for MPs mitigation due to their biodegradability and environmental safety. A genipin-cross-linked glutenin coating has shown excellent performance in reducing MPs shedding from plastic surfaces, even under harsh thermal, acidic, and saline conditions. Its self-adhesive nature, biocompatibility, and durability make it a promising preventive strategy at the source. Similarly, nanocellulose, derived from renewable biomass, exhibits a high surface area and tunable surface chemistry, making it effective for adsorbing MPs from water. Its natural abundance, low toxicity, and biodegradability position it as sustainable option for water treatment applications [129–130].

Another challenge that arises is the precise detection and characterization of MPs. Commonly employed techniques for MPs identification include spectroscopy, visual inspection, and thermal analysis. Among these, visual methods offer speed and simplicity; however, they often rely on personal judgment and are generally limited to the detection of larger particles [131]. Thermal analysis methods like thermogravimetric analysis/differential scanning calorimetry, pyrolysis gas chromatography mass spectrometry, and thermal extraction desorption gas chromatography are useful in identifying the chemical composition of MPs but are destructive analytical techniques [132–133]. Spectroscopic analysis like Raman spectroscopy often suffers from a poor signal-to-noise ratio and is affected by surface fluorescence from the samples, making careful sample preparation essential before analysis [134].

In recent years, advancements in MPs research have led to the development of innovative analytical tools for their detection, such as digital holography, scanning electron microscopy combined with energy dispersive X-ray spectroscopy, and terahertz time domain spectroscopy. These modern approaches enhance both accuracy and efficiency of detection compared to conventional methods. Nonetheless, they often involve complex sample preparation steps, which may result in cross-contamination or unintended loss of MPs particles during processing [135]. Moreover, these techniques face difficulties in accurately quantifying complex or heterogeneous samples, particularly when plastic particles are mixed with contaminants or display a typical colour [136]. While these advanced methods provide detailed and reliable data, their widespread use in large-scale and rapid environmental assessments is restricted due to their high costs, the need for careful sample preparation, and operational complexity. Conventional techniques for detecting MPs are often slow, require significant manual effort, and offer limited coverage. As a result, incorporating artificial intelligence (AI) and ML presents a promising solution by enhancing the speed, accuracy, and efficiency of MPs detection, making it a valuable tool in addressing the global challenge of MPs pollution [137].

**Figure 13:**
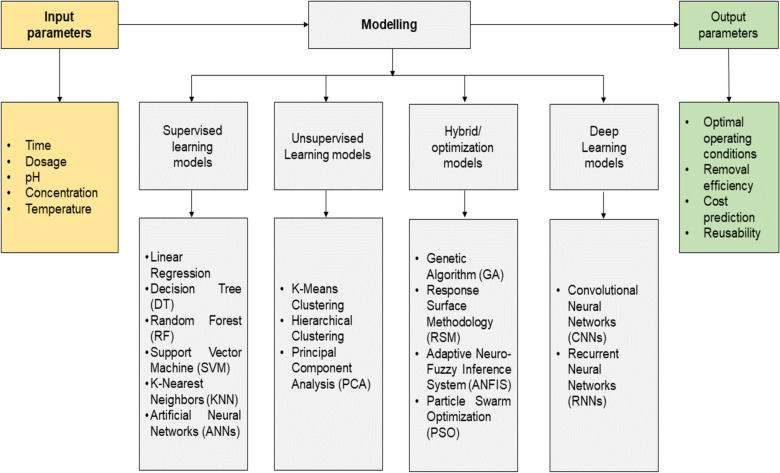
Different types of AI tools used for the detection of MPs.

Figure 13 provides a conceptual framework illustrating the different tools of AI and ML used in detection, classification, source identification, risk assessment, and management of MPs. Supervised learning algorithms, including linear regression, decision trees, random forests, support vector machines, k-nearest neighbours, and artificial neural networks, are widely used for predictive modelling. In contrast, unsupervised learning approaches such as k-means, spectral clustering, and mean shift are employed to identify the inherent patterns and groupings in datasets without predefined output variables, aiding in material classification or pattern recognition [138]. Hybrid and optimization based techniques including response surface methodology (RSM), particle swarm optimization (PSO), recurrent neural networks (RNN), and adaptive neuro-fuzzy inference systems (ANFIS) are useful in handling complex data types and fine tuning process parameters for improved performance [139]. Recent advancements in computational modeling and AI have significantly contributed to the development of more efficient and targeted strategies for MP and NP removal. As illustrated in Figure 13, AI and ML models serve as powerful tools for interpreting experimental data, identifying non-linear relationships among process variables, and predicting optimal treatment conditions.

Recent advancements in micro/nanorobotics have opened up new possibilities for the active removal of environmental contaminants, including MPs and NPs. These autonomous or externally guided micromachines can be engineered with functional surfaces that enable targeted adsorption, degradation, or collection of plastic particles from water bodies. For example, recent developments have demonstrated magnetically or chemically propelled microrobots capable of navigating complex aqueous environments to capture dispersed pollutants. Their high surface area-to-volume ratio, controllability, and potential for functionalization make them promising candidates for selective and efficient remediation. While still largely at the proof of concept stage, micro/nanorobotic systems represent a futuristic and highly adaptable approach to addressing micro/nanoplastic contamination [127].

In general, the future of MP detection depends on developing, with the help of AI, a standardized framework that integrates all stages, starting from data collection, to the analysis and risk evaluation. Current sampling and pre-processing methods face limitations such as complexity, inconsistent outputs, and time inefficiency, which intelligent sampling tools and cloud-based platforms aim to overcome through real-time optimization. Automated systems will enhance reagent selection and streamline workflows, increasing recovery rates during sample preparation. AI-assisted detection will combine traditional analytical techniques with ML to extract relevant features, eliminate background noise, and improve identification accuracy. In the risk assessment stage, AI will enable dynamic, real-time evaluation by integrating environmental and toxicity data, replacing outdated static methods. Ultimately, the use of AI will enhance the effectiveness of MPs monitoring and support the creation of data-driven environmental policies [134].

## Conclusion

MPs have emerged as persistent and pervasive pollutants in aquatic environments, with the potential to adsorb toxic substances, persist over long durations, and bio-accumulate through trophic levels. Their widespread detection in surface water, wastewater, and even drinking water systems has raised serious concerns about ecological and public health risks. While conventional water and wastewater treatment methods provide partial mitigation, particularly for larger particles, they often fall short in effectively removing nano- and microscale plastics. This review highlighted the growing relevance of nanotechnology in enhancing MPs remediation. Nanomaterials when employed as adsorbents, photocatalysts, or membrane modifiers, offer improved surface functionality, high reactivity, and tunable interactions with target pollutants. Their integration into treatment systems has demonstrated high efficiency under laboratory conditions, particularly in improving removal performance and selectivity toward various plastic types and associated co-contaminants. However, the transition from laboratory-scale experiments to real-world implementation faces several challenges. Key among them are the scalability of nanomaterial production, energy and cost demands, potential environmental and health concerns related to nanoparticle release, and the limited performance validation in complex, real water matrices. Additionally, the absence of standardized methods for assessing MP removal efficiency, as well as the environment safety of engineered nanomaterials, continues to hinder broader adoption and regulatory development. Addressing these gaps requires a multifaceted approach.

Future research must focus on environmentally benign and scalable synthesis methods, including the use of renewable feedstocks or waste-derived precursors. The development of robust, regenerable, and multifunctional nanomaterials that balance performance with environmental safety is critical. Furthermore, combining nanotechnology with existing treatment processes and integrating renewable energy sources, such as solar-driven photocatalysis, may offer pathways toward more sustainable operation. The application of machine learning and artificial intelligence for process optimization, predictive modelling, and real-time control is also a promising area that could accelerate the design and deployment of nano-enabled systems. The establishment of regulatory frameworks and standardized protocols for toxicity testing and environmental risk assessment of nanomaterials to ensure safe deployment is also equally important. Additionally, comprehensive life cycle assessments and techno-economic evaluations are necessary to assess the environmental impact, cost-effectiveness, and feasibility of nanotechnology based systems at scale. Collectively, these strategies will help to advance nanotechnology from the research domain into practical, safe, and sustainable solutions for MPs remediation in wastewater environments.

In summary, nanotechnology holds considerable promise in addressing the complex challenge of MPs pollution. While current advancements show case high removal efficiencies and material innovations, practical deployment will require continued interdisciplinary collaboration, life-cycle assessments, and system-level integration. As global demand for clean water intensifies, nanotechnology-driven approaches, if developed responsibly and sustainably, are poised to play central role in the future of microplastic remediation.

## Data Availability

Data sharing is not applicable as no new data was generated or analyzed in this study.
